# Supplementary material for: Pedagogical control scales of vertical jumping performance in untrained adolescents (13–16 years): research by strata
Source: PeerJ. 2024 Jun 17;12:e17298. doi: 10.7717/peerj.17298 (PMC11188930; doi:10.7717/peerj.17298)
Supplement: Supplemental Information 1 [file peerj-12-17298-s001.zip › CODEBOOK.docx]

**CODEBOOK**

1. ***Alpha K Folder:*** (Krippendorff's alpha):

- OriginalData: Includes a representative sample of vertical jump data (n=348) measured by ISAK specialists.
- Expert1: It includes the independent data of Expert 1 in his measurements of the vertical jump, to compare concordance.
- Expert2: It includes the independent data of Expert 2 in his measurements of the vertical jump, to compare concordance.

1. ***Descriptive Folder:*** Descriptive data collected

- CategoryFEM.13_14: Includes data from the 13-14 Female category
- CategoryFEM.16_16: Includes data from the 15-16 Female category
- CategoryMAS.13_14: Includes data from the 13-14 Male category
- CategoryMAS.15_16: Includes data from the 15-16 Male category

1. ***Ethnicity Folder:*** Data on ethnicity

- Ethnicity.FEM.ALL: Includes all data on the vertical jump, female gender (regardless of strata)
- Ethnicity.FEM.Strata: Includes all the data for the female gender in the vertical jump, classified by strata (1=Mongrel; 2=Montubio; 3=Afro-Ecuadorian; 4=Black; 5=Indigenous; 6=White; 7=Other)
- Ethnicity.MAS.ALL: Includes all data on the vertical jump, male gender (regardless of strata)
- Ethnicity.MAS.Strata: Includes all the data for the male gender in the vertical jump, classified by strata (1=Mongrel; 2=Montubio; 3=Afro-Ecuadorian; 4=Black; 5=Indigenous; 6=White; 7=Other)
- Ethnicity.FEM.13_14: Includes the vertical jump data in the female 13–14-year category.
- Ethnicity.FEM.13_14.Strata: Includes vertical jump data in the female 13-14 year-old category, classified by strata (1=Mongrel; 2=Montubio; 3=Afro-Ecuadorian; 4=Black; 5=Indigenous; 6=White; 7=Other)
- Ethnicity.MAS.13_14: Includes the vertical jump data in the male 13–14-year category
- Ethnicity.MAS.13_14.Strata: Includes vertical jump data in the male 13-14 year-old category, classified by strata (1=Mongrel; 2=Montubio; 3=Afro-Ecuadorian; 4=Black; 5=Indigenous; 6=White; 7=Other)
- Ethnicity.FEM.15_16: Includes the vertical jump data in the female 15–16-year category
- Ethnicity.FEM.15_16.Strata: Includes vertical jump data in the female 15-16 year-old category, classified by strata (1=Mongrel; 2=Montubio; 3=Afro-Ecuadorian; 4=Black; 5=Indigenous; 6=White; 7=Other)
- Ethnicity.MAS.15_16: Includes the vertical jump data in the male 15–16-year category
- Ethnicity.MAS.15_16.Strata: Includes vertical jump data in the male 15-16 year-old category, classified by strata (1=Mongrel; 2=Montubio; 3=Afro-Ecuadorian; 4=Black; 5=Indigenous; 6=White; 7=Other)

1. ***Percentiles-Gender Folder:*** Data on percentiles

- FEM.Jump.13_14: Includes the vertical jump of the category 13-14 years, female gender.
- FEM.Strata.13_14: Includes vertical jumps in the 13-14 years category, female gender, stratified by age (1=13 years; 2=14 years)
- MAS.Jump.13_14: Includes the vertical jump of the category 13-14 years, male gender.
- MAS.Strata.13_14: Includes vertical jumps in the 13-14 years category, male gender, stratified by age (1=13 years; 2=14 years)
- FEM.Jump.15_16: Includes the vertical jump of the category 15-16 years, female gender.
- FEM.Strata.15_16: Includes vertical jumps in the 13-14 years category, female gender, stratified by age (1=13 years; 2=14 years)
- MAS.Jump.15_16: Includes the vertical jump of the category 15-16 years, male gender.
- MAS.Strata.15_16: Includes vertical jumps in the 13-14 years category, male gender, stratified by age (1=13 years; 2=14 years)
- Gender.ALL13_14: Includes vertical jumps in the 13-14 years category of both genders.
- Gender.ALL13_14Strata: Stratifies the vertical jump data, category 13-14 years, by gender (1=female; 2=male)
- Gender.ALL15_16: Includes vertical jumps in the 15-16 years category of both genders.
- Gender.ALL15_16Strata: Stratifies the vertical jump data, category 15-16 years, by gender (1=female; 2=male)

1. ***Ranges Age Folder:*** Compare the age ranges (13-14 and 15-16 years old)

- CategoryFEM.13_14_15_16: Includes the female vertical jump data by categories (13-14 and 15-16 years)
- CategoryFEM.13_14_15_16.Strata: Stratifies the vertical jump data, category 13-14 and 15-16 years; (1=female; 2=male)
- CategoryMAS.13_14_15_16: Includes the male vertical jump data by categories (13-14 and 15-16 years)
- CategoryMAS.13_14_15_16.Strata: Stratifies the vertical jump data, category 13-14 and 15-16 years; (1=female; 2=male)

1. ***Socioeconomic Indicator Folder:*** Compare the results of the vertical jump according to socioeconomic indicators

- FEM13_14: Includes the vertical jump data of the category 13-14 years female
- Groups.Class.FEM13_14: Classifies the vertical jump data of the female 13-14 years category according to socioeconomic stratum (1=Upper class; 2=Middle class; 3=Lower class)
- FEM15_16: Includes the vertical jump data of the category 15-16 years female
- Groups.Class.FEM15_16: Classifies the vertical jump data of the female 15-16 years category according to socioeconomic stratum (1=Upper class; 2=Middle class; 3=Lower class)
- MAS13_14: Includes the vertical jump data of the category 13-14 years male
- Groups.Class.MAS13_14: Classifies the vertical jump data of the male 13-14 years category according to socioeconomic stratum (1=Upper class; 2=Middle class; 3=Lower class)
- MAS15_16: Includes the vertical jump data of the category 15-16 years male
- Groups.Class.MAS15_16: Classifies the vertical jump data of the male 15-16 years category according to socioeconomic stratum (1=Upper class; 2=Middle class; 3=Lower class)
- Female: Includes the vertical jump data of the category 13-14 and 15-16 years female
- Groups.Class.Female: Classifies the vertical jump data of the female 13-14 and 15-16 years category according to socioeconomic stratum (1=Upper class; 2=Middle class; 3=Lower class)
- Male: Includes the vertical jump data of the category 13-14 and 15-16 years male
- Groups.Class.Male: Classifies the vertical jump data of the male 13-14 and 15-16 years category according to socioeconomic stratum (1=Upper class; 2=Middle class; 3=Lower class)

1. ***Urban and Rural Zone:*** Compare the results of the vertical jump according to geographical area where they live (urban and rural)

- Zone.FEM.ALL: Includes all the female vertical jump data
- Zone.FEM.ALL.Strata: Includes all female vertical jump data, stratified by geographic area (1=Urban; 2=Rural)
- Zone.MAS.ALL: Includes all the male vertical jump data
- Zone.MAS.ALL.Strata: Includes all male vertical jump data, stratified by geographic area (1=Urban; 2=Rural)
- Zone.FEM.13_14: Includes the female vertical jump data, category 13-14 year
- Zone.FEM.13_14.Strata: Includes the female vertical jump data, category 13-14 year, stratified by geographic area (1=Urban; 2=Rural)
- Zone.MAS.13_14: Includes the male vertical jump data, category 13-14 year
- Zone.MAS.13_14.Strata: Includes the male vertical jump data, category 13-14 year, stratified by geographic area (1=Urban; 2=Rural)
- Zone.FEM.15_16: Includes the female vertical jump data, category 15-16 year
- Zone.FEM.15_16.Strata: Includes the female vertical jump data, category 15-16 year, stratified by geographic area (1=Urban; 2=Rural)
- Zone.MAS.15_16: Includes the male vertical jump data, category 15-16 year
- Zone.MAS.15_16.Strata: Includes the male vertical jump data, category 15-16 year, stratified by geographic area (1=Urban; 2=Rural)

**Note:** the subtitles are the rows names of each database with SPSS (option: variable view), with a description of their functions.
